# Gut Dysbiosis and IL-21 Response in Patients with Severe COVID-19

**DOI:** 10.3390/microorganisms9061292

**Published:** 2021-06-13

**Authors:** Mahejibin Khan, Bijina J. Mathew, Priyal Gupta, Garima Garg, Sagar Khadanga, Ashish Kumar Vyas, Anirudh K. Singh

**Affiliations:** 1 Microbiology and Fermentation Technology Department, CSIR-Central Food Technology Research Institute, Mysuru 570020, India; 2 Department of Microbiology, All India Institute of Medical Sciences, Bhopal 462024, India; bijina.micro@aiimsbhopal.edu.in (B.J.M.); priyal.phd2019@aiimsbhopal.edu.in (P.G.); garimadubey85@gmail.com (G.G.); ashishkvyas.microbiology@aiimsbhopal.edu.in (A.K.V.); 3 Department of Medicine, All India Institute of Medical Sciences, Bhopal 462024, India; sagar.genmed@aiimsbhopal.edu.in

**Keywords:** COVID-19, mucosal immunity, dietary fibre, gut microbiome, brain–gut interaction, IL-21

## Abstract

Background: The disease severity, ranging from being asymptomatic to having acute illness, and associated inflammatory responses has suggested that alterations in the gut microbiota may play a crucial role in the development of chronic disorders due to COVID-19 infection. This study describes gut microbiota dysbiosis in COVID-19 patients and its implications relating to the disease. Design: A cross sectional prospective study was performed on thirty RT-PCR-confirmed COVID-19 patients admitted to the All India Institute of Medical Sciences, Bhopal, India, between September 10 and 20, 2020. Ten healthy volunteers were recruited as the control group. IFN, TNF, and IL-21 profiling was conducted using plasma samples, and gut bacterial analysis was performed after obtaining the metagenomics data of stool samples. Results: Patients with a variable COVID-19 severity showed distinct gut microflora and peripheral interleukin-21 levels. A low Firmicute/Bacteroidetes ratio, caused by the depletion of the fibre-utilizing bacteria, *F. prausnitzii*, *B. Plebius*, and *Prevotella*, and an increase in Bacteroidetes has associated gut microbiota dysbiosis with COVID-19 disease severity. Conclusions: The loss of the functional attributes of signature commensals in the gut, due to dysbiosis, is a predisposing factor of COVID-19 pathophysiology.

## 1. Background

Coronavirus disease (henceforth COVID-19) is caused by SARS-CoV-2, which is one of the major pathogens that primarily targets the human respiratory system. The symptoms of COVID-19 infection appear after an incubation period of approximately 5.2 days and vary greatly [1]). Some people do not develop any symptoms and the infection is mild. In other patients, COVID-19 manifests as pneumonia and symptoms can range from fever to cough, dyspnea, shortness of breath, myalgia, fatigue, normal or decreased leukocyte counts, and gastrointestinal (GI) symptoms that damage digestive organs. These symptoms depend on the age of the patient and the status of the patient’s immune system [2]. Severe symptoms possibly occur due to a weak immune system that permits the faster progression of viral infection. Complications can also arise due to the release of cytokines during immune response and hyper inflammation or “cytokine storm syndrome” [3].

The largest component of the immune system, the mucosal surface, plays a key role in the inductive and effector phases of the mucosal response in the SARS-CoV-2 viral infection. Angiotensin-converting enzyme 2 (ACE2) receptor facilitate binding of SARS-CoV-2 through viral spike protein S, on epithelial cells causing the infection [4]. As ACE2 receptors are found in multiple organs, including the lungs, gut, and kidneys, and the finding that links the amino acid transport function of ACE2 to gut microbial ecology in the GI tract have suggested a link between COVID-19 and the enteric microbiota [5,6,7]. The detection of SARS-CoV-2 viral RNA in the faecal samples of COVID-19 patients has strengthened this hypothesis.

The human gut is home to trillions of beneficial bacteria. These microorganisms live in perfect harmony in GI tracts and produce active molecules, eliminate toxins, and educate the immune system to protect the host against harmful microbes [8,9]. The microbe–gut–brain axis explicitly includes the biochemical signalling of the central nervous system due to gut flora. The modulation of the endocrine system at the intestinal level is a type of neuro-entero-endocrine coordination that interacts with the immune system at the mucosal level in order to maintain homeostasis and enhance defence against various bacterial and viral pathogens. The intestinal mucosa is considered to be an immunological niche, as it hosts a complex immune-functional organ comprising T cell sub-populations and their related anti- and proinflammatory cytokines, as well as several other mediators of inflammation, in addition to trillions of commensal bacteria [10].

Respiratory viral infections may be associated with an altered gut microbiome and their correlation is known as the gut–lung axis. SARS-CoV-2 virus is reported in anal swabs and stool samples in almost 50% of patients with COVID-19, suggesting that the digestive tract might be an extrapulmonary site for virus replication and activity. In addition, common comorbidities related to the severity of COVID-19 are associated with modifications in bacteria taxa such as Bacteroidetes and Firmicutes, which are reported to regulate ACE2 expression in rodents [11,12,13].

Looking at the trends of the different levels of severity of COVID-19 infection, it has been speculated that the inflammatory responses in COVID-19 could be associated with gut microbiome variations; hence, to delineate the role of the gut microbiota in COVID-19, we studied the gut microbiota and cytokine profiles of COVID-19 patients and compared them with age matched non-diseased participants. 

## 2. Material and Methods

### 2.1. Study Design and Sample Collection

A cross sectional prospective study was performed on RT-PCR-confirmed COVID-19-infected patients admitted to the All India Institute of Medical Sciences, Bhopal, India, between September 10 and 20, 2020, for treatment. Healthy volunteers were recruited as a control group. The study was approved by the Institutional Human Ethics Committee of the All India Institute of Medical Sciences, Bhopal (IHEC-LOP/2020/EF0202). Informed consent was obtained from the patients and healthy volunteers before collecting the samples. Stool samples were self-collected by participants in sterile containers and kept in a refrigerator until they were processed for DNA isolation. No samples were kept in the refrigerator for more than 6 h. Blood was drawn in EDTA vials and plasma was separated by centrifugation at 250× *g* for 10 min. All the samples were collected at the time that patients were admitted to avoid the effect of drug treatment on the gut microbiota and serum levels.

### 2.2. Study Groups

The following four groups were recruited for the study: Group 1: adult healthy subjects found negative for SARS-CoV-2 infection through routine screening (control group). Group 2: asymptomatic SARS-CoV-2-positive patients with no symptoms, mostly health care workers or close contacts of known COVID-19 patients. Group 3: SARS-CoV-2-positive, with mild symptoms and no requirement for oxygen support/a ventilator. Group 4: SARS-CoV-2-positive with severe disease who were admitted to the AIIMS Bhopal and required oxygen support/a ventilator. 

### 2.3. Exclusion and Inclusion Criteria

Patients tested positive for SARS-CoV-2 in an RT-PCR diagnostic assay, and healthy subjects with a confirmed negative test for SARS-CoV-2 by an RT-PCR assay who were able to give informed consent were included in the study. Patients with co-infection and/or inability to give informed consent were excluded from the study.

### 2.4. Gut Microbiome Analysis

#### 2.4.1. DNA Extraction

DNA from stool samples was isolated using a NucleoSpin DNA Stool Mini Kit (MACHEREY-NAGEL GmbH & Co. KG, Dueren, Germany), as per the manufacturer’s protocol with some modifications. Briefly, a 180–220 mg stool sample was transferred to the tube containing ceramic beads. Lysis buffer was added and mixed by vortexing. The sample was heated at 70 °C for 5 min and lysed using Tissue LyzerLT (QIAGEN GmbH, Hilden, Germany) at 50 Hz for 10 min. The sample was processed as per the instructions and DNA was eluted in 30 µL of elution buffer.

#### 2.4.2. The Next-Generation Sequencing of Metagenomic DNA

Purified DNA was used to amplify 16 S rRNA hyper variable region V3-V4 using KAPA HiFiHotStart Ready Mix and modified 341 F and 785 R primers [14] and sequence libraries were prepared. 

#### 2.4.3. Sequence Data Processing and Statistical Analysis

Raw sequence data were filtered using Trimgalore [15] to remove low quality sequences and data quality was confirmed using Fast QC [16] and MultiQC software. The data were checked for Base call quality distribution, % bases above Q20, Q30, %GC, and sequencing adapter contamination. UCHIME algorithm [17] was used to check chimeric sequences. The raw sequence data that was generated were submitted under the bio project at NCBI.

The filtered contigs that passed the QC threshold (Q20 > 95%) were processed and classified into taxonomical outlines based on the GREENGENES v.13.8–99 database [18]. The contigs were then clustered into OTUs (Operational Taxonomic Units). After the classification, the OTU relative abundance was estimated. 

Alpha diversity, which is a measurement of the richness and relative abundance of bacteria within the sample, was assessed using the ACE, InvSimpson, and Fisher indices. Beta Diversity was analysed by the LDA Effect Size (LEfSe) to compare the microbial diversity between the control and test groups. We first used the non-parametric factorial Kruskal–Wallis (KW) sum-rank test to detect features with significant differential abundance with respect to the class of interest; biological significance was subsequently investigated using a set of pairwise tests among subclasses using the (unpaired) Wilcoxon rank-sum test. LEfSe uses Linear Discriminant Analysis to estimate the effect size of each differentially abundant feature.

The differences in the relative abundance of the gut microbial population among the groups was analysed using a Student’s- *t*-test at a 95% confidence level.

### 2.5. Quantification of Plasma IL-21, TNF-α, and INF-γ

The concentrations of IL-21, TNF-α, and INF-γ were measured in the plasma of all patient groups using the respective pre-coated CUSABIO kits following the manufacturer’s protocol. The lowest detection limit for IL-21 was 3.2 pg/mL.

## 3. Results

### 3.1. COVID-19-Infected Patients

The study included a total of 40 volunteers (*n* = 40); 30 patients tested positive for SARS-CoV-2 in an RT-PCR test. Ten healthy individuals found negative for SARS-CoV-2 in the RT-PCR test formed a control group (Table 1). The median age of COVID-19 patients was between 45.9 and 57.79 years. There were significant differences in lymphocyte count, neutrophil counts, and C-reactive proteins among the COVID-19 patients (*p* < 0.05).

### 3.2. Patients with Different Severity Levels Had Altered Gut Microbiota That Was Less Diverse

**Figure 1:**
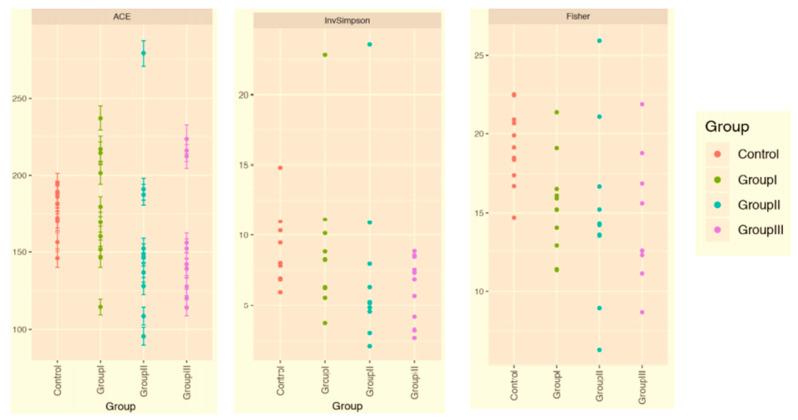
Alpha diversity of the gut microbiota of COVID-19 and healthy subjects. Control: non-COVID-19 subject; Group I: asymptomatic group; Group II: mildly infected group; Group III: severely infected group.

The gut bacterial microbiota of all the test groups was studied using a high-throughput next-generation sequencing of the V3–V4 region of the 16 S ribosomal RNA gene. The ACE, InvSimpson, and Fisher indices were used to analyse the richness and evenness of the bacterial species within each group. Bacterial gut microbial diversity was prominent in the control group. The diversity decreased with the increasing severity of symptoms, and the least diversity was observed in the group classified as ‘severe’ (Figure 1).

**Figure 2:**
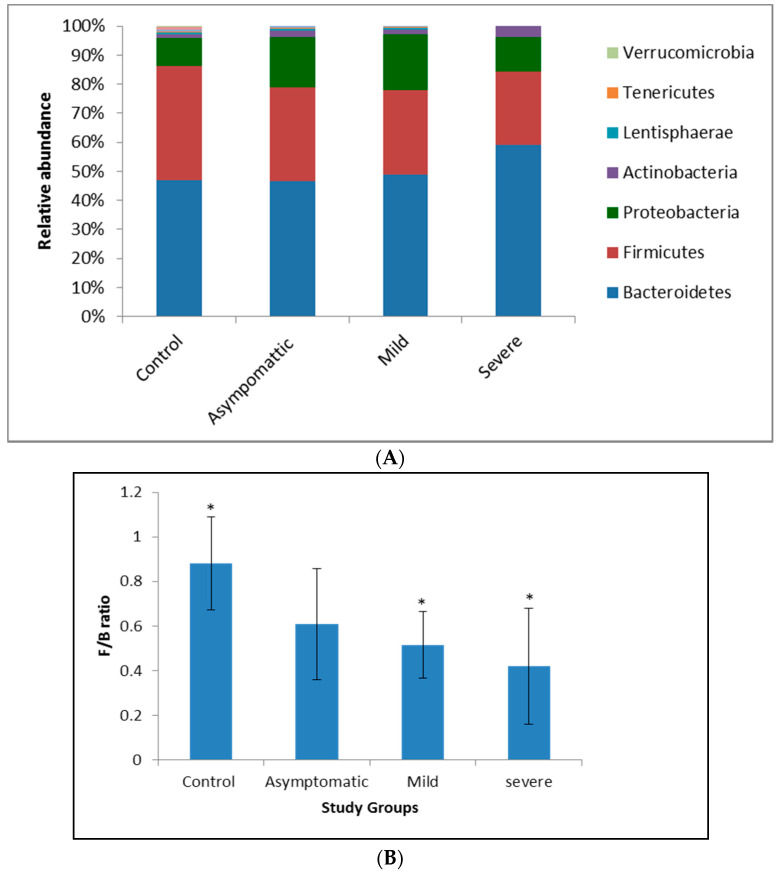
Compositional changes in the gut microbial diversity of COVID-19-infected and -noninfected subjects. Results are expressed as mean ± SD, *n* = 10. Statistical significance was determined with a Student’s *t*-test (unpaired, two tailed), * *p* = 0.05. (**A**): Relative abundance of microbial phyla in the gut of COVID-19-infected and non-infected groups; (**B**): Firmicutes/Bacteroidetes ratio in the gut of COVID-19-infected patients and noninfected groups.

To understand the variations in the gut bacterial diversity between the control and the diseased groups, a beta diversity analysis was carried out. Firmicutes and Bacteroidetes dominated at the phylum level, but the relative abundance of Firmicutes decreased significantly (*p* < 0.05) in the COVID-19-diseased groups as compared to the control. A reduction in Firmicutes by 15, 20, and 34 percent and an increase in the counts of Bacteroidetes by 2.41, 13.77, and 29.40 percent characterised the asymptomatic, mild, and severe patients, respectively (Figure 2A). The observed gradual decline in Firmicutes/Bacteroidetes (F/B) ratios in mild (0.51) and severe (0.42) patients (Figure 2B) contrasted with the higher F/B ratios of the healthy (0.89) and asymptomatic (0.61) participants. Similarly, Proteobacteria increased by 44% and Actinobacteria by 130% in mild and severely infected COVID-19 patients (Figure 2A).

### 3.3. Bacterial Genera and Species Differences in the Guts of COVID-19 Patients

**Figure 3:**
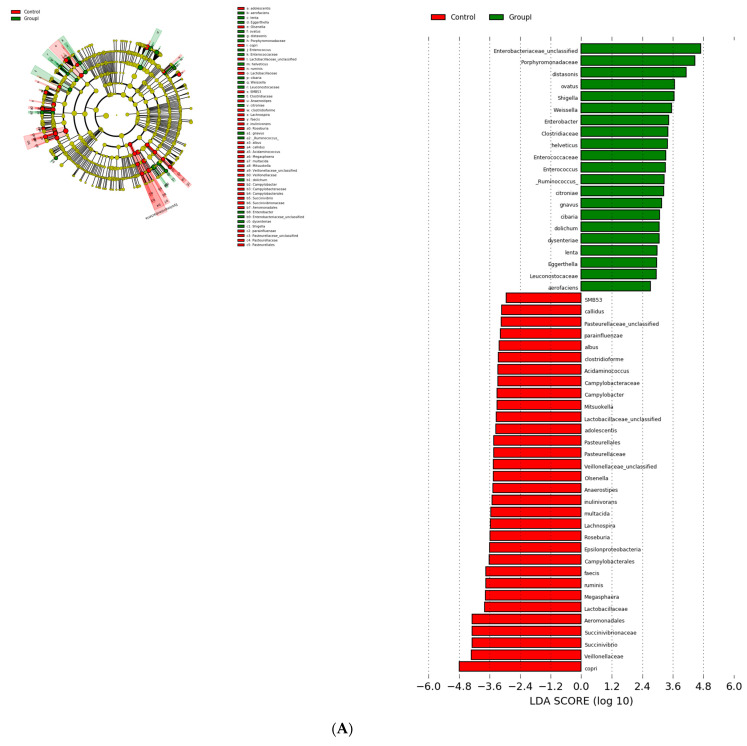

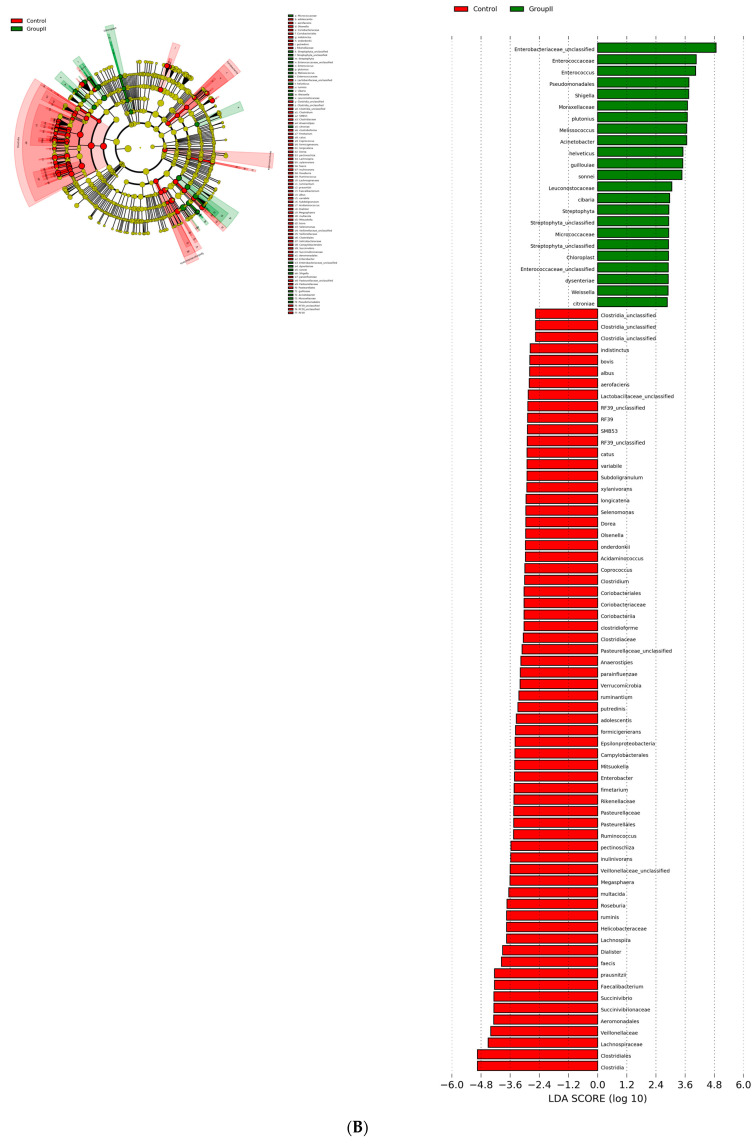

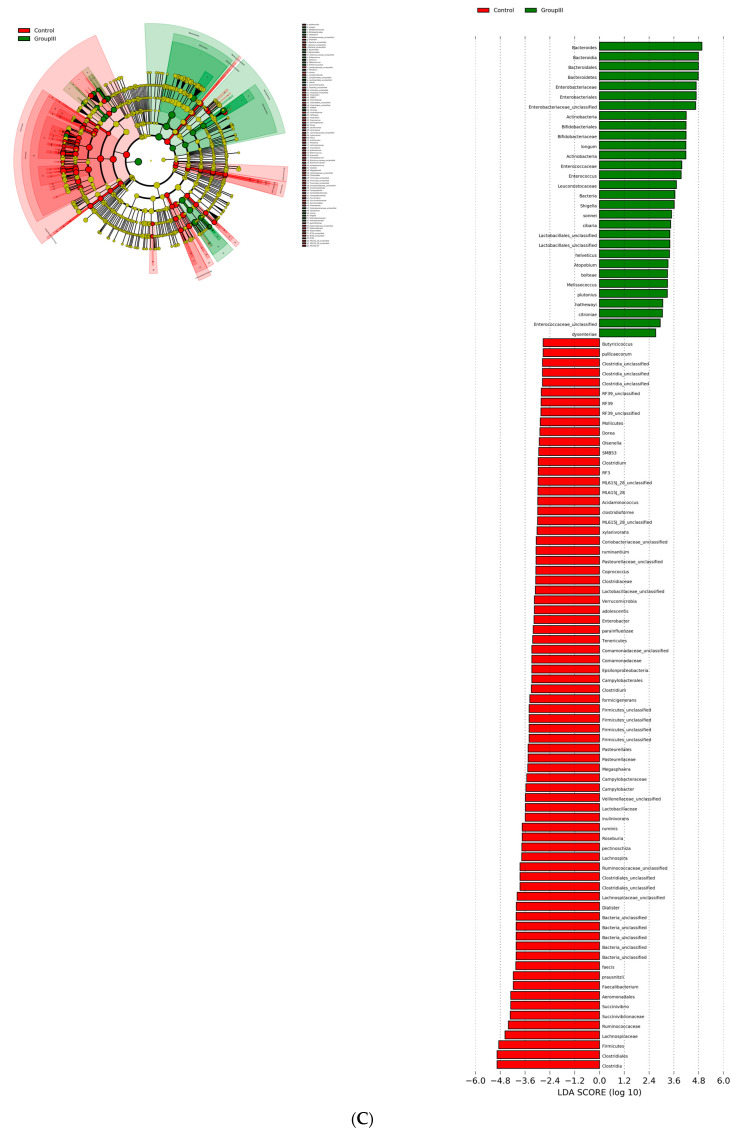
Cladograms and histograms showing the diversity of the gut microbiota in COVID-19-infected patients: (**A**) control vs. asymptomatic; (**B**) control vs. mild; (**C**) control vs. severe.

Alterations in the gut bacterial genera and species of COVID-19-infected patients were studied using a Linear Discriminant Analysis (LDA) effect size (LEfSe). A logarithmic LDA score cutoff of 2.0 was used to identify important taxonomic differences between the healthy and SARS-CoV-2-infected patients. There was a significant difference in bacterial groups at an LDA score (log10) > 2 between the healthy and COVID-19-infected patients (Table 2; Figure 3A–C).

**Figure 4:**
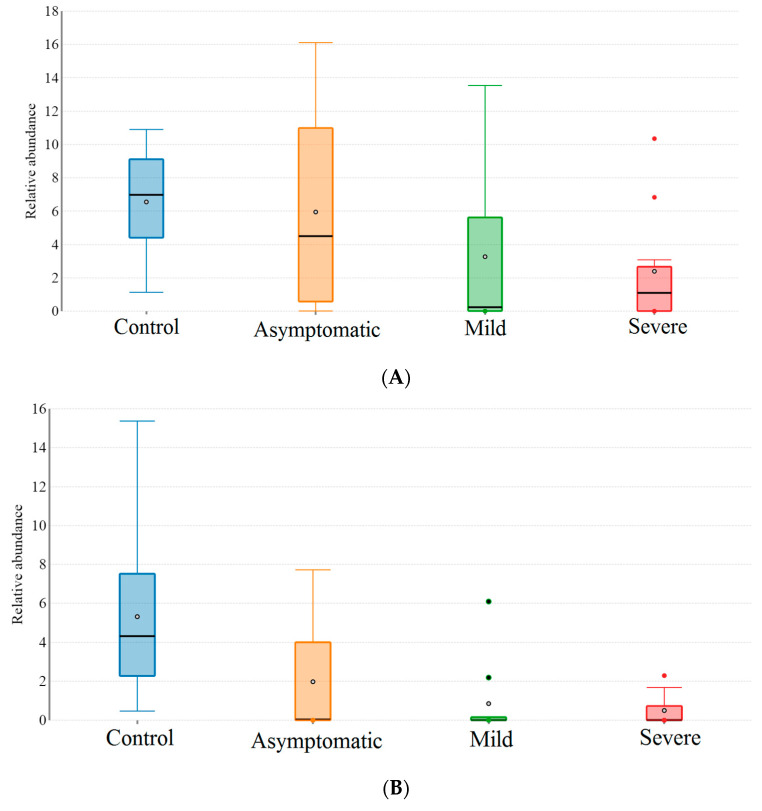

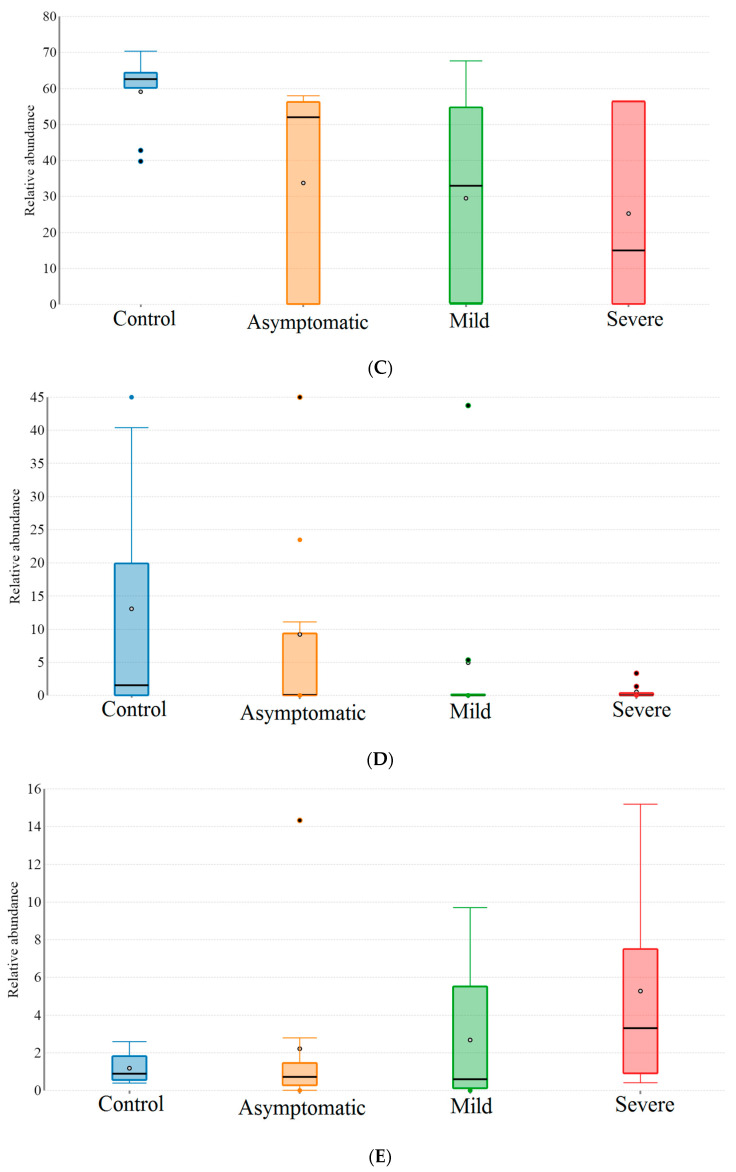
Box plot showing gut bacterial species abundance in healthy and COVID-19-infected groups. (**A**) *Faecalibacterium prausnitzii*; (**B**) *Roseburia* sp.; (**C**); *Prevotella* (**D**); *Bacteroides plebeius* (**E**) *Bifidobacterium*.

Beta diversity was analysed using Linear Discrimination Analysis (LDA) effect size (LEfSe). The *Lachnospiracece* and *Ruminococcceae*, families belonging to Clostridium clusters XIVa and IV dominated the healthy guts (i.e., the guts of the control group). The reduction in the *Lachnospiraceae* abundance (16% in the asymptomatic group, 28% in the mildly infected group, and 43% in the severely infected group) was similar to that observed with *B. plebeius*, *Prevetolla*, *F. prausnitzii*, and *Roseburia spp.* Some of the bacterial groups, such as *Butyricicoccus pullicaecorum*, *Clostridium ruminatium*, *Lachnospira pectinoschiza*, and *Pseudobutyrivibrio xylanivorans*, were completely absent in the guts of COVID-19-infected patients. However, they were enriched in *C. hathewayi*, *Parabacteroides distasonis*, and *R. gnavus*. The *Bifidobacterium* count increased fivefold in the severely infected group (Table 3 and Table 4; Figure 4).

### 3.4. Severe COVID-19 Patients Have Elevated Circulating IL-21 Levels

**Figure 5:**
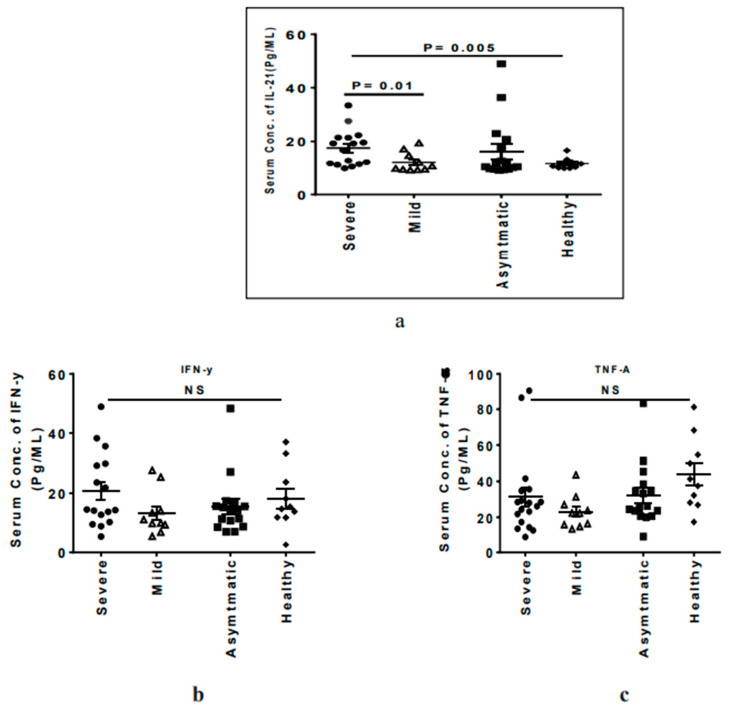
Dot plot graph showing the levels of different cytokines in the study groups: horizontal short bars indicate the mean ± standard deviation. *p* < 0.05 is considered significant. (**a**) IL-21, (**b**) IFN-γ, (**c**), TNF-α.

Inflammatory cytokines such as IFN-γ, TNF-α, and IL-21 have been shown to play a critical role in the severity of SARS-CoV-2-mediated infection [19]. In the present study, it was observed that the severely ill patients had higher levels of IL-21 compared to mildly ill (17.54 ± 6.76 vs. 12.13 ± 3.71, *p* = 0.01) and healthy participants (17.54 ± 6.76 vs. 11.6 ± 2.07, *p* = 0.005) (Figure 5a). However, we did not see any significant difference in the TNF-α and INF-γ levels among the groups (Figure 5b,c).

## 4. Discussion

The symbiotic gut microbiota, along with the host’s mucosal immune system, functions as the first line of defence against infectious organisms. It covers respiratory, gastrointestinal, and urogenital tracts and shapes the physiological immune response of the host by secreting signalling molecules and immunological markers. Imbalances in gut homeostasis (dysbiosis) can cause severe inflammatory responses that can even contribute to tissue damage [20,21].

During the current COVID-19 pandemic, the largest mortality and morbidity rates are reported in elderly groups and in patients previously diagnosed with chronic disorders such as obesity, coronary heart disease, or diabetes [22,23]. The detection of high levels of calprotectin in the faecal samples of patients has related the disease severity to the inflammatory responses of the intestine [24]. Alterations in the intestinal microbiota composition and their metabolites affect metabolic inflammation, thereby contributing to several health disorders [25]. Therefore, the relevance of the gut microbiome to COVID-19 infection and disease was investigated. A significant increase in Bacteroidetes (*p* < 0.05) caused by the progression of COVID-19 disease from an asymptomatic stage to a severe stage and a concomitant reduction in the relative abundance of Firmicutes (Table 3)—specifically, a low Firmicutes to Bacteroidetes ratio—is indicative of dysbiosis. Similar low Firmicutes to Bacteroidetes ratios have been reported in low-grade systemic inflammation, cognitive deficits, depression, Crohn’s disease, and type 2 diabetes [26,27]. In addition, consistent enrichment of Bacteroidetes in mild and severe COVID-19 patients has suggested the presence of an inflammatory immune response in these patients. Members of Bacteroidetes, *Bacteroides dorei*, and *B vulgaris* have been shown to stimulate macrophages and monocytes to secrete a complex array of pro-inflammatory cytokines, interleukin-1, CXCL8, IFNγ, and TNFα for aberrant inflammatory reactions due to cytokine storm [28]. While we did not observe any significant difference in the levels of IFNα and IFNγ in our study group, higher IL-21 levels in severe COVID-19 patients suggests the existence of a hyper inflammatory condition in this group. A similar increase in IL-21 levels and Bacteroidetes abundance together with a low Firmicutes/Bacteroidetes ratio as observed in the inflammatory disease Systemic Lupus erythematosus [29,30] supports this idea. These findings also suggest a direct relation of cytokine to the gut microbiome dysbiosis and an inflammatory response exacerbation of COVID-19 disease (Lucas et al., 2020).

The regulation of mucosal surface ACE2 expression, the receptor involved in COVID-19 infection, has been correlated with gut microbial composition—specifically to *Coprobacillus* and some of the *Bacteroidetes* species that upregulate colonic ACE2 expression [31]. Likewise, Firmicutes species have been reported to variably affect the expression of ACE-2 receptors [32,33]. Therefore, the differential abundance of Bacteroidetes species and low Firmicute counts in the COVID-19-diseased groups as compared to the control group observed in this study (Figure 3A) may explain distinctive dysbiosis to ACE-2 expression as determinants of COVID-19 disease severity.

The depletion of *B. plebeius* in severely diseased COVID-19 patients (0.58 as against 13.66 in healthy control participants) reflects a dampening of the immunoregulatory effects against inflammation and allergy [34,35]. The bacterium, characterized from the guts of Japanese natives, degrades seaweed porphyran and agarose into 3,6-Anhydro-L-galactose (AHG) and agaro-oligosaccharides [36,37], effecting an anti-inflammatory reaction through the inhibition of the MAPK and NF-κB pathways [38].

Proteobacteria is a sensitive phylum of gut microbiome and an indicator of unbalanced gut community [39]. Proteobacterial abundance (13.55 ± 8.89) in COVID-19-infected patients as against uninfected healthy participants (9.5 ± 7.8.5) correlated dysbiosis to the disease. Along with low Firmicutes counts, the data substantiated a role in cytokine overproduction and inflammatory responses [40,41,42].

The results describe COVID-19 infection influencing the colonic microbiota homeostasis by inciting the switching of coelomocyte metabolism. Such an alteration results in upset gut obligate anaerobic bacteria dominance, negatively affecting the benefit that the host generally derives by converting fibre into fermentation products. In this context, the study reveals a reduction in *Prevotella* numbers, affecting (a) the gut mucin glycoprotein maintenance, which interacts with the immune system, and (b) bringing down fibre fermentation, thereby affecting short-chain fatty acids, propionates, and butyrates production, leading to inflammatory responses. The reduced counts of *Lachnospira, Veillonella, Ruminococcus, Faecalibacterium*, and *Roseburia* belonging to Clostridium XIVa and IV clusters and decreased *Prevotella*, which dominates the gut microbiome of healthy and asymptomatic groups (Table 3), documents an affected gut microbiome homeostasis. The impaired biological functions result in inflammatory responses and colonocyte metabolism that maintain and shape the GI barrier [43].

Typical dysbiosis was the differential decrease in the Clostridium taxa, *F. prausnitzii* in the guts of the mildly and severely diseased COVID-19 patients (Figure 5). This important commensal protects the host from inflammation by blocking NF-κB activation and IL8 production [44]. It also regulates the expression of anti-inflammatory cytokines (IL-10, TGF-β2, and IL-1 Ra); down-regulates the expression of pro-inflammatory cytokines such as IL-6, TNF-α, and TNF-β; and inhibits the secretion of IL-6 through the reduced phosphorylation of JAK2/STAT3 [45,46]. The identification of the single bacterium suggests the development of a genetic tool that would enumerate their numbers for an assay that can forecast the COVID-19 disease and prognosis for severity.

Though higher counts of *Bifodobacterium* were recorded in severely ill COVID-19 patients, its functional significance was affected due to dysbiosis. The consequent lactate accumulation due to the reduction in Clostridiales and the depletion of fibre-degrading species, *Butyricicoccus pullicaecorum, C. ruminatium, Lachnospira pectinoschiza*, and *Pseudobutyrivibrio xylanivorans*, enforced another systemic inflammatory disorder in COVID-19 disease syndrome, similar to Behcet’s disease [47].

The main limitation of this study is the sample size; studies describing the relationship between the gut microbiome and COVID-19 disease severity need larger sample sizes. This trial study from an Indian population presents the influence of the gut microbiome composition and diversity on SARS-CoV-2 infection and disease severity. Factors such as comorbidities were adjusted and patients with coinfection were excluded from the study groups to control for the variations in the gut microbiota in response to these differences. Similarly, care was taken to not include patients with a known history of diabetes and other comorbidities linked to COVID-19 severity and gut dysbiosis, and all non-control participants were COVID-19-positive. However, in the absence of records of unsupervised medications received by patients before being admitted to AIIMS, Bhopal, it is difficult to rule out the effect of these medications, if any, on the gut microbiota. Nonetheless, it is plausible to assume that such factors will have a negligible effect on our analyses due to the small number of participants in each group. All the samples were collected from a particular region to control for gut microbiome variations due to dietary patterns. Study groups were categorised as mild and severe based on oxygen/ventilator requirement and symptom severity, including severity of fever and breathing trouble. Stool samples were collected at the time of admission to the hospital, and post recovery samples were not collected to avoid the drug-induced perturbation in the gut microbiome.

## 5. Conclusions

This study suggests that gut microbiome dysbiosis is an important predisposing factor for COVID-19 disease severity. The impaired gut homeostasis affected in fibre-utilizing bacteria deprived the cells lining the colon of energy, thereby making them susceptible to the virus. The changes may have actuated aberrant mucosal immunological reactions due to a Proteobacterial increase and Firmicutes decrease. The depletion of *B. plebeius* and *F. prausnitzii* in the guts further amplified the situation by causing cytokine overproduction and dysregulated inflammation.

Dysbiosis, reflecting the affected metabolome in the inflamed gut of COVID-19 patients due to reduced levels of fibre-utilizing bacteria, despite the high Bifidobacterial numbers, suggests the use of consortium-based therapy for homeostasis rather than a diet-based intervention through prebiotics and probiotics. Such therapy will also prevent robust shifts in the gut ecosystem that the administration of single-strain probiotics, specific prebiotics, or their combination may cause.

## Figures and Tables

**Table 1:** Clinical parameters of patients at the admission stage to AIIMS, Bhopal.

Parameters/Patient Group	Asymptomatic (Mean ± SD)	Mild (Mean ± SD)	Severe (Mean ± SD)	*p*-Value *
Age (years)	57.9 ± 17.99	49.4 ± 13.12	45.9 ± 8.82	0.34
Total Leukocyte Count (×10^9^/µL)	6927.78 ± 4342.4	10,481.11 ± 5772.13	6878 ± 3566.17	0.17
Neutrophils	58.56 ± 22.20	77.22 ±17.06	76 ± 12.42	0.27
Lymphocytes	32.11 ± 19.34	15.55 ± 13.28	18.67 ± 11.98	0.23
C reactive protein (mg/L)	11.41 ± 10.54	44.66 ± 31.76	99.18 ± 41.15	0.02
Eosinophil	1.56 ± 2.65	0.5 ± 1.06	0.4 ± 0.89	0.65
Aspartate aminotransferase (U/L)	41.08 ± 24.34	54.62 ± 28.63	54.47 ± 31.59	0.78
Alanine aminotransferase (U/L)	53.74 ± 78.56	66.17 ± 58.76	53.97 ± 41.72	0.76

* *p*-value was calculated using one-way ANOVA.

**Table 2:** Changes in the relative operational taxonomic units of the gut microbiota.

S.No	Comparison	OTU (abs LDA Score > 2.0)
1	Healthy vs. asymptomatic	53
2	Healthy vs. mildly infected	89
3	Healthy vs. severely infected	104

**Table 3:** Change in the Mean Relative Abundance of fibre-degrading bacterial species in the guts of COVID-19-infected patients. Bacterial phyla and species were identified in the faeces after next-generation sequencing. Reference control is the phyla and species found in healthy guts.

Phylum	Species	Percent Reduction in Mean Relative Abundance		
		COVID-19 Infected		
		Asymptomatic	Mild	Severe
Bacteroidetes	* Bacteroides plebeius	29.3	62.2	96.6
Firmicutes	*Faecalibacterium prausnitzii*	3.32	27.9	50.59
Firmicutes	*Roseburia faecis*	54.70	77.24	85.87
Firmicutes	*Roseburia inulinivorans*	85.2	41.52	96.17
Firmicutes	*Dorea formicigenerans*	30.12	69.87	61.44
Firmicutes	*Lachnospira pectinoschiza*	61.29	77.71	96.77
Firmicutes	*Pseudobutyrivibrio xylanivorans*	50.0	Absent(100)	Absent(100)
Firmicutes	*Clostridium ruminantium*	59.09	Absent(100)	Absent(100)
Firmicutes	*Butyricicoccus pullicaecorum*	40	90.09	Absent(100)
		**Percent increase in Mean Relative abundance**		
Actinobacteria	*Bifidobacterium* sp	87.61	126.43	347.24

* Agarose and Porphyran degrading gut bacteria.

**Table 4:** Bacterial groups positively associated with severely infected COVID-19 patients.

Phylum	Species #	Mean Relative Abundance (%)		*p*-Value
		Control	COVID-19	
Bacteroidetes	*Bacteroides caccae*	1.35	6.35	0.003
Bacteroidetes	*Bacteroides ovatus*	0.22	1.85	0.0284
Bacteroidetes	*Parabacteroides distasonis*	0.736	7.887	0.376
Bacteroidetes	*Bacteroides fragilis*	0.015	3.36	0.002
Firmicutes	*Ruminococcus gnavus*	0.021	1.96	0.135
Firmicutes	*Clostridium bolteae*	2.29	3.29	0.036
Firmicutes	*Clostridium citroniae*	0.02	0.888	0.013
Firmicutes	*Clostridium hathewayi*	0.001	0.971	0.03
Proteobacteria	*Shigella sonnei*	1.95	3.48	0.0052
Proteobacteria	*Shigella dysenteriae*	0.001	0.33	0.022
Actinobacteria	*Atopobium rimae*	4.18	4.63	0.0303

**#** Taxa ordered to by log discriminative analysis scored by LDA size effect.

## Data Availability

Gut Metagenome raw Sequence data were deposited to the National Centre for Biotechnology Information Sequence Read Archive under BioProject accession number PRJNA705797. Patients’ data and other details are available with the corresponding authors.
